# Dysregulated m6A-Related Regulators Are Associated With Tumor Metastasis and Poor Prognosis in Osteosarcoma

**DOI:** 10.3389/fonc.2020.00769

**Published:** 2020-06-02

**Authors:** Jianhao Li, Benchen Rao, Jie Yang, Liwen Liu, Maoxin Huang, Xin Liu, Guangying Cui, Chao Li, Qicai Han, Hao Yang, Xichun Cui, Ranran Sun

**Affiliations:** ^1^Precision Medicine Center, The First Affiliated Hospital of Zhengzhou University, Zhengzhou, China; ^2^Key Laboratory of Clinical Medicine, The First Affiliated Hospital of Zhengzhou University, Zhengzhou, China; ^3^Department of Orthopedics, Zhengzhou Central Affiliated Hospital to Zhengzhou University, Zhengzhou, China; ^4^Dermatology Department, The First Affiliated Hospital of Zhengzhou University, Zhengzhou, China; ^5^Department of Orthopedics, The First Affiliated Hospital of Zhengzhou University, Zhengzhou, China; ^6^Department of Bone and Soft Tissue, The Affiliated Cancer Hospital of Zhengzhou University, Zhengzhou, China

**Keywords:** N6-methyladenosine, osteosarcoma, tumor metastasis, prognosis, biomarkers

## Abstract

**Background:** Osteosarcoma (OS) is the most common primary bone tumor. The disease has a poor prognosis due to the delay in the diagnosis and the development of metastasis. N6-Methyladenosine (m6A)-related regulators play an essential role in various tumors. In this study, a comprehensive analysis was conducted to elucidate the relationship between the expression profiles of m6A-related molecules and the clinical outcome of OS patients.

**Materials and Methods:** Public genome datasets and a tissue microarray (TMA) cohort were used to analyze the mRNA and protein expression levels of m6A regulators. Next, immunofluorescence (IF) analysis was used to determine the subcellular localization of m6A-related molecules. Kaplan–Meier and Cox regression analyses were performed to confirm the prognostic value of m6A-related molecules in OS. A comprehensive bioinformatic analysis was conducted to identify the potential molecular mechanisms mediated by m6A modification in OS.

**Results:** We found that m6A-related regulator expression was dysregulated in OS tissues, especially in metastatic tumor tissues. Low expression of METTL3, METTL14, and YTHDF2 and high expression of KIAA1429 and HNRNPA2B1 were significantly associated with poor prognosis in the TMA cohort. Simultaneously, the genome meta-cohort analysis revealed that low expression of FTO and METTL14 and high expression of METTL3, HNRNPA2B1, and YTHDF3 were associated with poor prognosis in OS. Cox regression analysis showed that HNRNPA2B1 might be an independent risk factor for OS. Bioinformatic analysis indicated that m6A regulators might be involved in OS progression through humoral immune response and cell cycle pathways.

**Conclusion:** M6A-related regulators are frequently dysregulated and correlate with metastasis and prognosis in OS. M6A-related regulators may serve as novel therapeutic targets and prognostic biomarkers for OS.

## Introduction

Human osteosarcoma (OS) is one of the most common aggressive bone cancers, and it has higher incidence and mortality rates in teenagers; it frequently affects distal femur, proximal tibia, and humerus (1, 2). Despite significant progress in therapeutic strategies against OS over the past few decades, its prognosis remains poor due to the delay in the diagnosis and the development of metastasis (3). Thus, it is critical to clarify novel biomarkers and to ensure the effective treatment of OS patients.

N6-Methyl adenosine (m6A) is a posttranscriptional modification of RNA and is the most prevalent internal chemical modification of mRNAs (4). M6A network components have been well characterized into three subtypes: “writers,” “readers,” and “erasers” (5). The m6A modification is facilitated by writers and removed by erasers; moreover, it can recruit specific reader proteins (6). M6A-related regulators are involved in various physiological and pathological processes through the regulation of RNA stability, mRNA splicing and translation, and microRNA processing (7–9). Additionally, m6A is involved in the initiation and progression of cancers, including liver cancer, breast cancer, glioma, cervical cancer, colorectal cancer, and hepatoblastoma (10–15). However, the clinical value and potential mechanism of m6A-related regulators in OS are still unclear.

In this study, we evaluated the expression status and prognostic value of m6A-related proteins in OS based on public genome database analysis and tissue microarray (TMA) analysis. We found that dysregulated expression of several m6A-related proteins was frequently and closely associated with tumor metastasis and clinical outcomes in OS. Moreover, consensus clustering for m6A-related regulators was performed to identify the clusters with a better prognosis. Mechanistically, Gene Set Enrichment Analysis (GSEA) and Gene Set Variation Analysis (GSVA) indicated that the dysregulated m6A-related regulators might be associated with cell cycle and humoral immune response pathways. In conclusion, we revealed that m6A-related regulators play a critical role in the development of OS and proposed that m6A-related regulators could be potential therapeutic targets.

## Materials and Methods

### Datasets

The RNA-seq transcriptome data and clinical information from four OS datasets (GSE12865, GSE21257, GSE42352, GSE39055, TARGET-OS) were collected from the GEO (http://www.ncbi.nlm.nih.gov/geo/) and TARGET (https://ocg.cancer.gov/programs/target) databases, based on the inclusion and exclusion criteria of the GEO and TARGET databases. Inclusion criteria: datasets involving human osteosarcoma and expression profiling by array. Exclusion criteria: datasets with a sample size smaller than 10 (2, 11, 16, 17). The characteristics of the datasets are presented in Table S1. Subsequently, three gene expression matrix files (GSE21257, GSE39055, TARGET-OS) with survival follow-up data were merged into one meta-cohort. Next, the “sva” package of the R software was used to remove the batch effect (Figure S1). A total of 175 patients with survival follow-up information were divided into higher and lower expression groups using the best cutoff value of the log-rank test. Kaplan–Meier survival curves were used to analyze survival rates. Univariate and multivariate analyses were conducted based on the Cox model. The raw data were analyzed by BRB-array tools as previously described (18).

### Tissue Samples

Studies involving human samples were approved (approval number: 2011-KY-047) by the Ethics Committee of the First Affiliated Hospital of Zhengzhou University (ZZU cohort), Zhengzhou Central Affiliated Hospital of Zhengzhou University, and the Affiliated Cancer Hospital of Zhengzhou University. All legal guardians of the children signed an informed consent. OS samples from the three hospitals were combined to create the TMA cohort containing 120 OS tissues and 65 surrounding non-tumorous tissues.

### Immunohistochemistry (IHC)

Immunohistochemistry (IHC) of m6A-related genes was performed as described previously (19, 20). Briefly, 5-μm-thick TMA sections were deparaffinized and then treated with hydrogen peroxide to quench endogenous peroxidase activity. Subsequently, the sections were incubated overnight with anti-human m6A-related protein antibodies (1:200, Abcam, USA) at 4°C. Next, the immunoreactive cells were detected by SignalStain® DAB (CST, USA), then counterstained with Haematoxylin QS (Vector Laboratories). Two experienced pathologists, who were blinded to the clinicopathological data, evaluated the immunostaining samples separately. A semi-quantitative scoring system was established based on different staining intensities, and the proportion of positive cells was scored as follows: 0, none; 1+, <25%; 2+, 25–50%; 3+, 50–75%; and 4+, 75–100%. The staining intensity was scored as follows: 0, none; 1+, weak; 2+, medium; and 3+, strong. The total score was calculated by multiplying two subscores, and the samples with scores of 0–6 were regarded as low expression, whereas the other ratings were regarded as high expression during statistical analysis. Antibody information is listed in Table S2.

### Cell Lines and Culture

The human OS cell lines U2-OS and KHOS-240S were purchased from ATCC (Manassas, USA). Cells were maintained at 37°C in a humidified atmosphere of 5% CO_2_ in Dulbecco's modified Eagle medium (DMEM) supplemented with 10% fetal bovine serum (FBS) (Gibco, New York, NY, USA) and 100 U/mL penicillin/streptomycin (Corning, New York, NY, USA). The STR reports for the cell lines U2-OS and KHOS-240S are presented in Supplemental Materials 1–2.

### Immunofluorescence Assay

OS cells cultured in 24-well plates were fixed in 4% paraformaldehyde and permeabilized in 1% Triton X-100 phosphate-buffered saline (PBS). After blocking with 1% bovine serum albumin (BSA), the cells were incubated with primary antibodies at 4°C overnight and then with appropriate corresponding secondary antibodies (Jackson ImmunoResearch Inc., USA) at room temperature for 30 min. Finally, the nuclei were counterstained with DAPI (Beyotime, China), and images were obtained with a Zeiss Axio microscope (Zeiss, Oberkochen, Germany). Detailed information on the antibodies used in this study is listed in Table S2.

### Statistical Analysis

All statistical analyses were performed using the R statistical package (R version 3.5.3) unless otherwise stated. The differences between the two independent groups were analyzed using Student's *t* test (unpaired, two-tailed) or Permutation test when there were fewer than three samples in either group (21). Kaplan–Meier overall survival analysis was performed with a log-rank test. Univariate Cox regression analysis was used to indicate the relationship between the different variables and survival. The correlation was evaluated using the two-tailed Pearson test. We clustered OS patients into different clusters with “ConsensusClusterPlus” (22). Additionally, 150 clinically actionable genes were obtained from a recent publication (23). Subsequently, the protein–protein interactions among m6A regulators and 150 clinically actionable genes were identified based on the STRING (https://string-db.org/) interaction database (24). Cytoscape software was used to visualize the interactions. GSVA was performed using the Bioconductor R package “GSVA” (25). GSEA was conducted using clusterProfiler, an R/Bioconductor package (26). In all cases, *P* < 0.05 was considered statistically significant expression is frequently dysregulated.

## Results

### M6A-Related Gene Expression Is Frequently Dysregulated in Osteosarcoma

**Figure 1:**
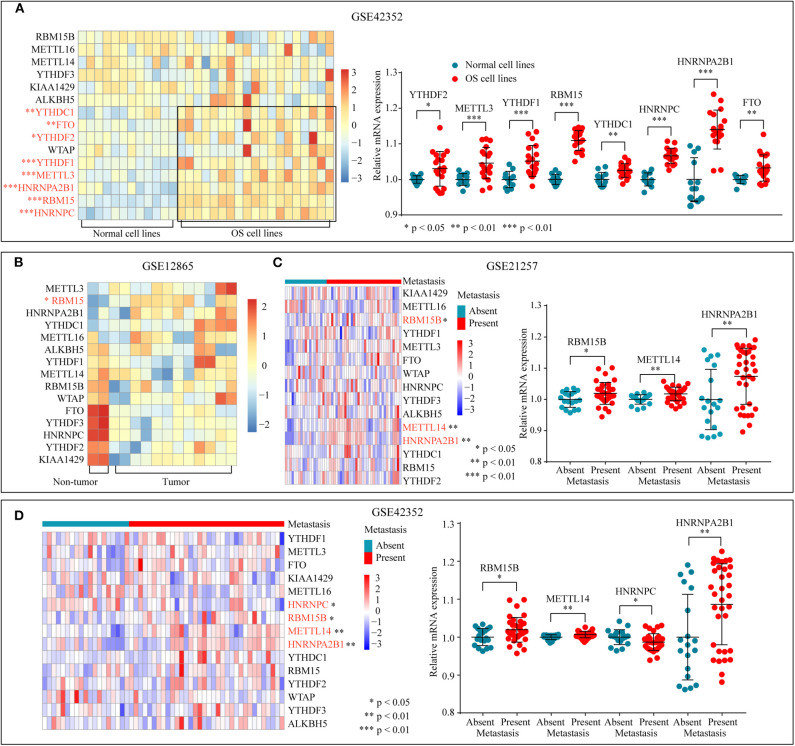
m6A-related regulators expression status in osteosarcoma. **(A)** The mRNA expression level of m6A-related regulators in normal and OS cell lines of GSE42352. **(B)** The mRNA expression level of m6A-related molecules in tumor and non-tumor tissues of GSE12865. **(C,D)** The correlation between the mRNA expression of m6A-related regulators and tumor metastasis.

To determine the significant biological function of m6A-related regulators in tumorigenesis and development, the expression pattern of m6A-related genes was analyzed through the GEO database (GSE42352) (Figure 1A). The mRNA expression levels of YTHDF2, YTHDF1, HNRNPC, FTO, METTL3, RBM15, HNRNPA2B1, and YTHDC1 were higher in OS cells than in normal cell lines. In addition, differential expression analysis of GSE12865 through a permutation test further confirmed that the expression of RBM15 was significantly upregulated in the tumor tissues (Figure 1B). Subsequently, the relationship between tumor metastasis and the expression level of m6A-related regulators was examined in GSE21257 and GSE42352 (Figures 1C,D). The overexpression of RBM15B, METTL14, and HNRNPA2B1 is significantly related to tumor metastasis. In conclusion, these results suggested that dysregulated m6A-related regulators were associated with tumorigenesis and metastasis in OS.

### Subcellular Location of m6A-Associated Proteins in Osteosarcoma Cell Lines

**Figure 2:**
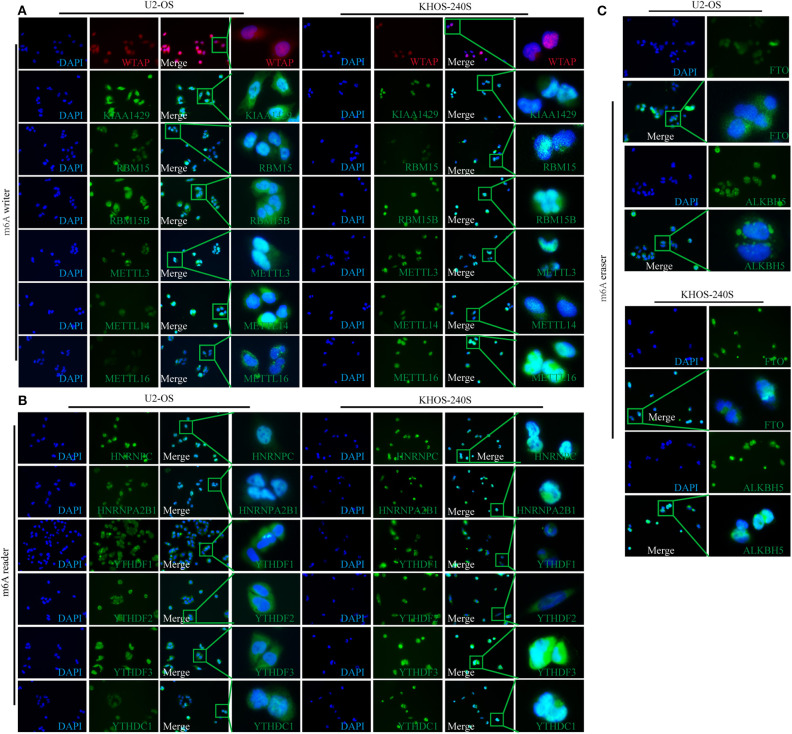
Subcellular location of m6A-related molecules in two osteosarcoma cell lines. **(A)** Immunofluorescence of m6A “writers” in U2-OS and KHOS-240S cell lines. **(B)** Immunofluorescence of m6A “readers” in U2-OS and KHOS-240S cell lines. **(C)** Immunofluorescence of m6A “erasers” in U2-OS and KHOS-240S cell lines.

The diverse subcellular location of proteins may reflect on the different functions of m6A-related regulators in OS cells. Therefore, IF was performed to determine the subcellular location of m6A-related proteins in U2-OS and KHOS-240S cell lines. We found that all the m6A “writers” had intense nuclear and weak cytoplasmic staining in the two OS cell lines (Figure 2A). Moreover, m6A “readers” HNRNPC and HNRNPA2B1 were detected only in the nucleus, whereas YTHDF1 and YTHDF2 had weak nuclear and intense cytoplasmic staining. The fluorescence signal of YTHDF3 was intense in the nuclear and cytoplasm while the staining intensity of YTHDC1 was weak in the cytoplasm and nucleus (Figure 2B). As m6A methylation erasers, FTO and ALKBH5 were moderately expressed in the cytoplasm and nucleus (Figure 2C). The details of the subcellular localization of the m6A-related proteins in OS cells were presented in Table S3.

### Dysregulated m6A-Related Regulators Are Associated With Poor Prognosis in Osteosarcoma

**Figure 3:**
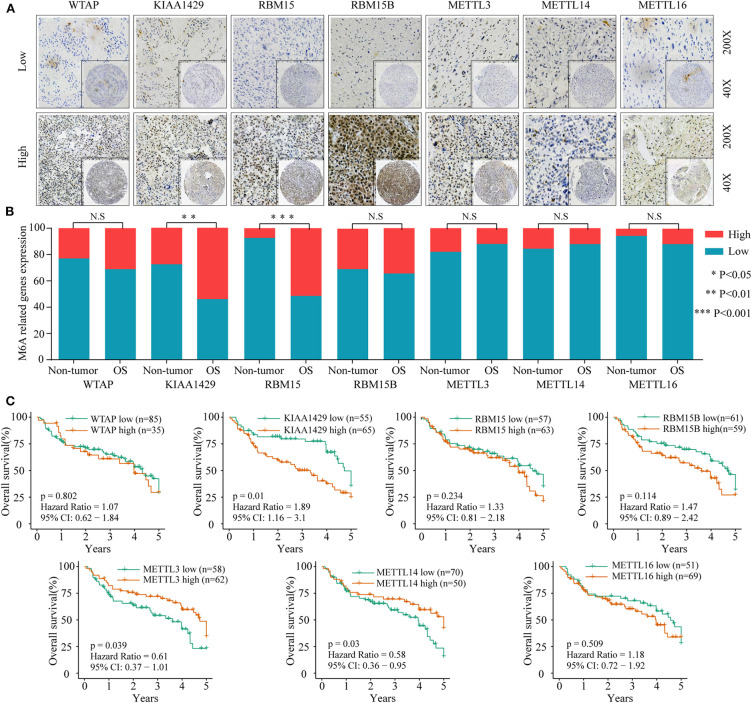
The relationship between the expression level of m6A-related “writers” and poor prognosis in osteosarcoma. **(A)** Representative IHC high and low staining of m6A “writers” in OS tissues. **(B)** Comparison of m6A-related “writers” relative expression between OS tissues and nontumor tissues in TMA-combined cohort. **(C)** Kaplan–Meier analysis of the correlation between m6A “writers” expression and overall survival of OS patients in the TMA-combined cohort.

**Figure 4:**
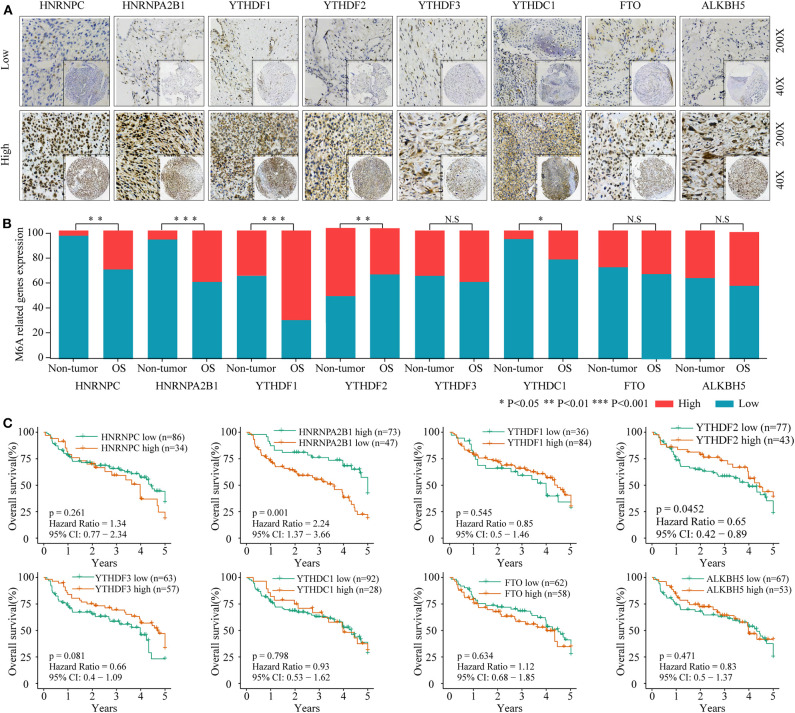
The relationship between the expression level of m6A related “reader” and “erasers” and poor prognosis in osteosarcoma. **(A)** Representative IHC high and low staining of m6A “reader” and “erasers” in OS tissues. **(B)** Comparison of m6A “reader” and “erasers” relative expression between OS tissues and non-tumor tissues in combined-TMA cohort. **(C)** Kaplan–Meier analysis of the correlation between m6A “reader” and “erasers” expression and overall survival of OS patients in the TMA-combined cohort.

To investigate the association between m6A-related protein expression and the clinical outcome of OS patients, a TMA cohort containing 120 OS tissues and 65 surrounding non-tumorous tissues was employed (Figures 3A, 4A). Differential expression analysis indicated that the protein expression levels of KIAA1429, RBM15, HNRNPC, HNRNPA2B1, YTHDF1, YTHDF2, and YTHDC1 were higher in tumor samples than in non-tumor samples (Figures 3B, 4B). Moreover, osteosarcoma patients were divided into two groups (high and low expression groups) based on each m6A-related protein IHC staining intensity and survival analysis was performed. The results revealed that high expression of KIAA1429 and HNRNPA2B1 was significantly associated with poor overall survival rates, while low expression of METTL3, METTL14, and YTHDF2 was related to poor prognosis in OS (Figures 3C, 4C).

**Figure 5:**
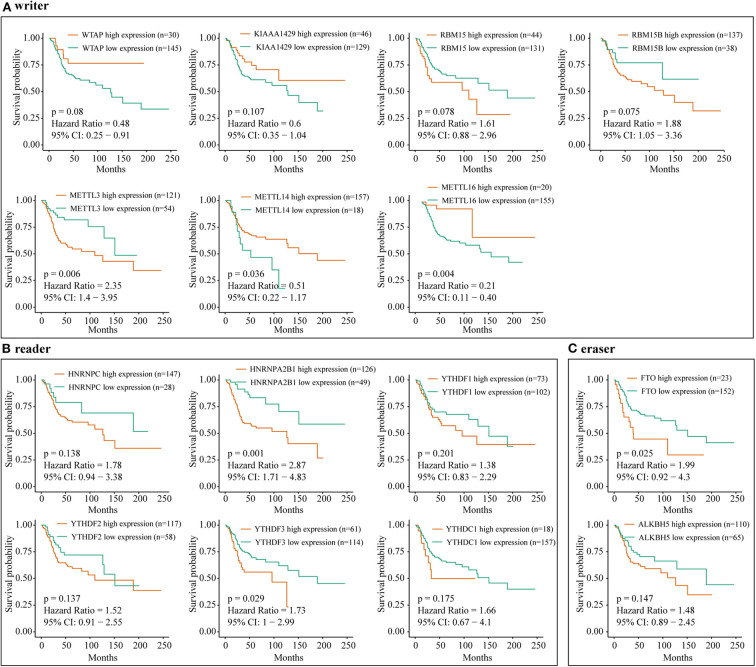
The correlation between m6A-related regulator expression and overall survival of osteosarcoma patients in the meta-cohort. **(A)** Kaplan–Meier analysis showing the correlation between m6A “writers” expression levels and the OS patients' survival rates. **(B)** Kaplan–Meier analysis showing the correlation between m6A “readers” expression levels and the OS patients' survival rates. **(C)** Kaplan–Meier analysis showing the correlation between m6A “erasers” expression levels and the OS patients' survival rates.

**Figure 6:**
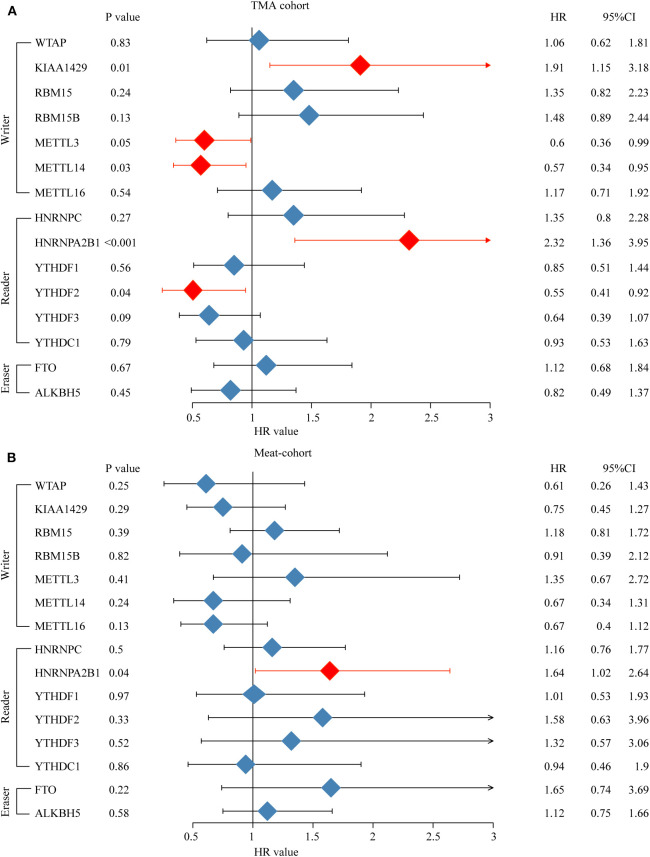
High expression level of HNRNPA2B1 is correlated with poor prognosis of osteosarcoma. **(A)** Univariate Cox regression analysis of OS patients' overall survival in meta-cohort. **(B)** Univariate Cox regression analysis of OS patient's overall survival in TMA combined OS cohort.

Furthermore, an OS genome meta-cohort containing 175 OS tissues was constructed based on three independent datasets (GSE21257, GSE39055, and TARGET-OS, Figure S1). Overall survival analysis was performed, and the results indicated that the patients with high expression of METTL3, HNRNPA2B1, YTHDF3, and FTO and low expression of METTL14 had a significant shorter OS rate (Figures 5A–C). Furthermore, univariate analysis based on the TMA cohort suggested that the high expression of KIAA1429 and HNRNPA2B1 and the low expression of METTL3 and METTL14 were potential independent prognostic risk factors for OS patients. Moreover, the prognostic value of HNRNPA2B1 was validated in the genome meta-cohort, and KIAA1429, METTL3, and METTL14 did not exhibit significant prognostic potential (Figures 6A,B). In conclusion, dysregulated m6A-related regulators may predict poor prognosis in OS.

### A Cluster Associated With a Favorable Prognosis Was Identified Through Consensus Clustering of m6A-Related Genes

To further explore the prognostic value of m6A-related gene expression patterns and to understand their underlying mechanisms in OS, we analyzed the relationships among 15 m6A-related regulators. Then, we identified a cluster associated with favorable outcomes and explored its possible mechanisms based on the genome meta-cohort.

**Figure 7:**
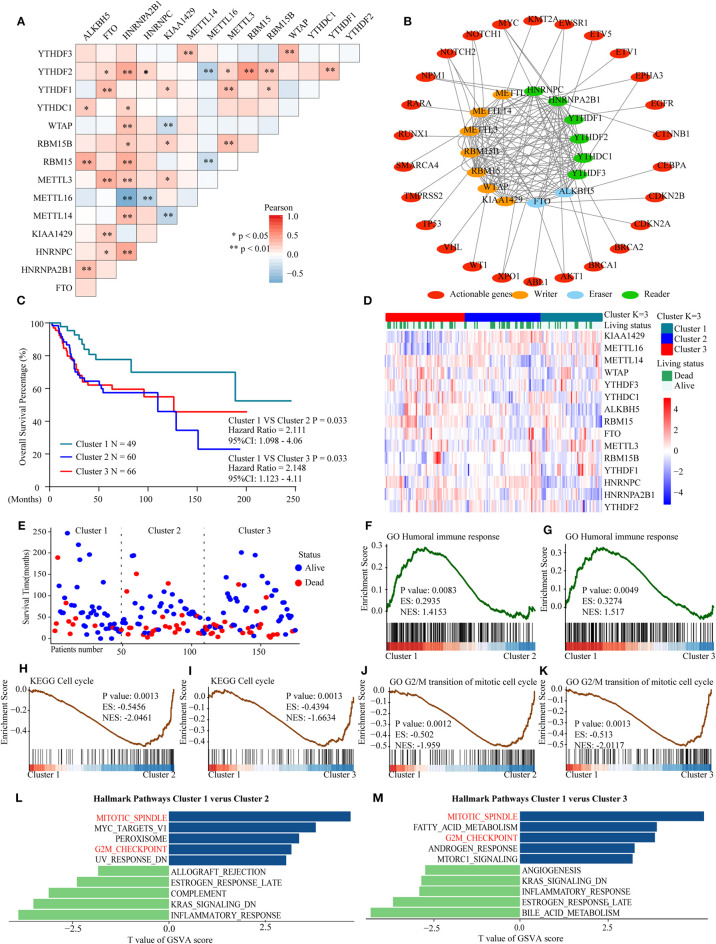
Interaction among m6A RNA–related regulators, differential survival status, and functional annotation of OS in three clusters. **(A)** Pearson correlation analysis of the 15 m6A regulators. **(B)** Protein–protein interactions among m6A regulators and clinically actionable genes obtained from the STRING database. **(C)** Kaplan–Meier overall survival curves for 175 osteosarcoma patients. **(D)** Heatmap and survival status of the three clusters defined by the m6A related regulators' consensus expression. **(E)** Three-cluster scatter plot of different survival time and status. **(F,G)** GSEA revealed that genes with higher expression in the cluster 1 subgroup were enriched for humoral immune response. **(H,I)** GSEA revealed the relationship between three clusters in osteosarcoma and cell cycle pathway. **(J,K)** GSEA revealed the relationship between three clusters in osteosarcoma and transition of the mitotic cell cycle pathway. **(L,M)** GSVA among three clusters using hallmark gene sets.

Considering the similarity of the biological functions of m6A-related regulators, we analyzed the correlation (Figure 7A) and interaction (Figure 7B) among the 15 m6A-related genes. The results indicated that there were higher correlations among HNRNPA2B1, YTHDF2, RBM15, and ALKBH5. However, there was an inverse correlation between METTL16 and HNRNPA2B1, YTHDF2, and RBM15 expression in OS. Moreover, the protein–protein interaction networks showed that these m6A-related proteins interacted with each other frequently (Figure 7B). Additionally, the interaction between m6A-related proteins and 150 clinically actionable genes was examined to understand the clinical significance of m6A-related proteins. The results showed that m6A-related proteins frequently interacted with some of these genes (Figure 7B). Conclusively, the biological functions of 15 m6A-related genes were closely related in OS.

Furthermore, three subgroups were clustered by consensus clustering based on the expression of m6A-related genes in the genome meta-cohort. Patients were divided into three clusters with K = 3. Survival analysis showed that cluster 1 had a significantly longer overall survival than cluster 2 and cluster 3 (Figures 7C–E). Subsequently, GSEA revealed that the humoral immune response was significantly enriched in cluster 1 which exhibited good prognosis (Figures 7F,G), while the cell cycle and G2/M transition of mitotic cell cycle pathways were significantly enriched in cluster 2/3 which exhibited poor prognosis (Figures 7H–K). Similarly, the results of GSVA indicated that the dysregulated m6A-related genes might promote mitotic spindle and G2M checkpoint pathways, thus resulting in poor prognosis in OS (Figures 7L,M). Overall, these findings indicated that m6A modification may be involved in OS progression by regulating the humoral immune response and cell cycle–related pathways in OS.

## Discussion

Many studies have demonstrated that dysregulated m6A modification is associated with human diseases such as obesity, type 2 diabetes mellitus, and infertility (27–29). In addition, the dysregulated expression of m6A-related genes is closely related to multiple tumors, including OS. To date, there have been only two reports addressing the functional role of m6A in osteosarcoma. Wang et al. demonstrated that METTL3 and ALKBH5 were closely associated with doxorubicin resistance in OS (30). Miao et al. reported that upregulated METTL3 promoted osteosarcoma progression by regulating LEF1 (31). However, the expression status and functional role of m6A-related genes in tumorigenesis and progression of OS have not yet been systematically analyzed.

**Figure 8:**
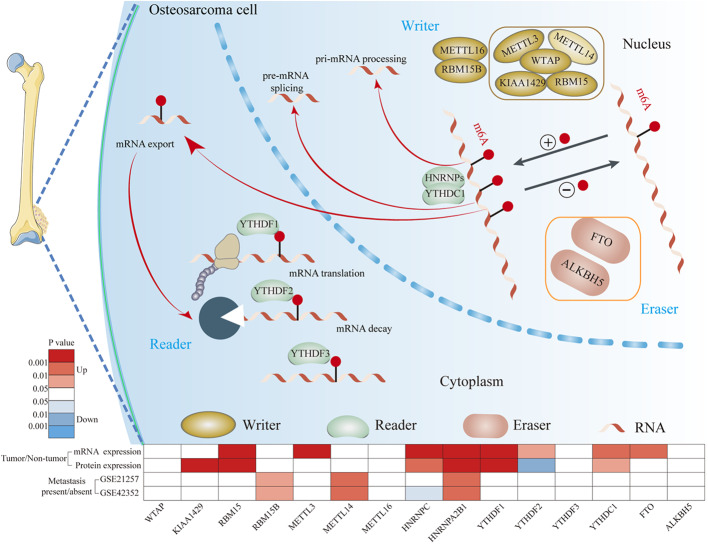
The mechanism of m6A modification in osteosarcoma cells. m6A of RNA methylation is dynamically regulated by binding proteins “writers” (WTAP, KIAA1429, RBM15, RBM15B, METTL3, METTL14, and METTL16), “readers” (YTHDF1, YTHDF2, YTHFDF3, YTHDC1, and HNRNPs) and “erasers” (FTO and ALKBH5). “writers” add m6A modification on RNA, “readers” are proteins that bind to m6A modifications and exert various functions including pre-mRNA splicing, pri-miRNA processing, nuclear export, RNA translation modulation and mRNA decay. “erasers” could serve as demethylases.

In this study, we comprehensively analyzed the mRNA and protein expression levels of m6A-related regulators between OS and normal tissues based on two relatively large-scale OS cohorts (Figure 8). Both cohorts confirmed that the expression levels of RBM15, HNRNPC, HNRNPA2B1, YTHDF1, and YTHDC1 were increased in OS tissues. Consistent with our results, previous studies have demonstrated that RBM15 is upregulated in chronic myelogenous leukemia (32). HNRNPC and HNRNPA2B1 are upregulated in breast cancer (33, 34). YTHDF1 is upregulated in ovarian cancer, lung cancer, hepatocellular carcinoma, and colorectal cancer (35–38). YTHDC1 expression is increased in Kaposi's Sarcoma (39). In general, these findings suggest that m6A-related regulators are frequently dysregulated in OS. These dysregulated m6A-related regulators may be involved in the tumorigenesis and progression of OS.

Numerous studies have reported that dysregulated m6A-related molecule expression is closely associated with the outcome of tumor patients (23). Nevertheless, the impact of m6A methylation on OS prognosis is still unclear. For the first time, we systematically analyzed the prognostic value of m6A-related regulators in OS at both on the mRNA and protein levels. We found that high expression of HNRNPA2B1 and low expression of METTL14 were significantly associated with poor overall survival in the genome meta-cohort. Similarly, upregulated HNRNPA2B1 and downregulated METTL4 were positively related to low survival rates in the TMA cohort. Consistent with our results, HNRNPA2B1 has been reported to act as an oncogene in breast cancer, pancreatic cancer, lung cancer, hepatocellular carcinoma, and cervical cancer (34, 40–43), while METTL14 functions as a suppressor gene in glioma and breast cancer (44, 45). In contrast, the potential role of METTL14 as an oncogene has also been observed in acute myelocytic leukemia and hepatocellular carcinoma (44, 46). In summary, the expression pattern of m6A-related regulators may be considered a potential prognostic biomarker in OS.

M6A-related regulators can play a crucial role in cancer progression by targeting downstream molecules in an m6A-dependent manner. For instance, METTL3 promotes tumor progression by regulating LEF1 and SOCS2 (13, 31); ALKBH5 promotes tumor cell proliferation by increasing FOXM1 (47); YTHDF1 silences the drug-resistance gene AKR1C1 (36); and KIAAL429 targets and downregulates GATA3, which further contributes to liver cancer progression (48). To explore the underlying mechanism involved in m6A modification–mediated OS progression, we performed a systematic bioinformatic analysis based on an OS genome meta-cohort. Frequent cross talk among m6A-related regulators was observed in OS. Subsequently, we identified three clusters by consensus clustering based on the expression of m6A-related genes and found that cluster 1 had a significantly better prognosis than the others. Bioinformatic analysis revealed that cluster 1 was closely associated with the activated humoral immune response and suppressed cell cycle–related pathways. Aberrant regulation of the immune response and cell cycle–related pathways is considered a primary causative node in cancer progression (49, 50). Interestingly, recent literature has suggested that m6A modification can regulate the cell cycle and humoral immune response pathways (51, 52). It is therefore not surprising that the potential regulatory network between m6A modification and immune response or cell cycle–related pathways, which need further investigation.

However, our present study has some shortcomings. First, we only analyzed the relationship between dysregulated m6A-related regulators and clinical features in OS but did not verify these findings through *in vivo* and *in vitro* experiments. Second, we identified that cluster 1 was associated with a better prognosis. However, these findings need further validation in more independent cohorts. Third, we only explored the underlying mechanism based on bioinformatic prediction. M6A modification–mediated aberrant activation or suppression pathways in OS remains a subject for further study.

## Conclusion

In summary, we have identified for the first time that m6A-related regulators are frequently dysregulated and closely correlated with poor survival in OS. M6A modification–mediated aberrant activation of cell-cycle related pathways and suppression of immune response may be responsible for the crucial role of m6A in OS progression. These findings may present a promising diagnostic biomarker and a potential target therapeutic strategy for OS patients.

## Data Availability Statement

Publicly available datasets were analyzed in this study. This data can be found here: GEO http://www.ncbi.nlm.nih.gov/geo/, TARGET /https://ocg.cancer.gov/programs/target.

## Ethics Statement

The studies involving human participants were reviewed and approved by the Ethics Committee of the First Affiliated Hospital of Zhengzhou University, Zhengzhou Central Affiliated Hospital of Zhengzhou University, and the Affiliated Cancer Hospital of Zhengzhou University. Written informed consent to participate in this study was provided by the participants' legal guardian/next of kin.

## Author Contributions

JL and MH performed all the experimental work. JY, CL, and HY collected osteosarcoma specimens. QH and XL participated in data analysis. RS, XC, and GC conceived and participated in the design of the study. The manuscript was written by JL and BR. All authors read and approved the final manuscript.

## Conflict of Interest

The authors declare that the research was conducted in the absence of any commercial or financial relationships that could be construed as a potential conflict of interest.
