# Supplementary material for: Dysregulated m6A-Related Regulators Are Associated With Tumor Metastasis and Poor Prognosis in Osteosarcoma
Source: Front Oncol. 2020 Jun 2;10:769. doi: 10.3389/fonc.2020.00769 (PMC7280491; doi:10.3389/fonc.2020.00769)
Supplement: Supplementary file 1 [file Table_1.docx]

**Table S1. Datasets information used in this study.**

| Datasets | Platforms | Non-tumor samples | Tumor samples | Metastasis | Treatment regimens | Follow-up information | | Year | Country |
| --- | --- | --- | --- | --- | --- | --- | --- | --- | --- |
| GSE12865 | Affymetrix | 2 | 12 |  |  |  | | 2009 | Canada |
| GSE42352 | Illumina | Mesenchymal stem cells 12  Human Osteoblast 3 | Osteosarcoma cell line 19  Osteosarcoma biopsies 84 | Yes 34  No 19 | MAP-based chemotherapy |  | | 2012 | Norway |
| GSE21257 | Illumina | **0** | 53 | Yes 34  No 19 | MAP-based chemotherapy | Overall survival | | 2012 | Norway |
| GSE39055 | Illumina | **0** | 37 |  | MAP-based chemotherapy | Overall survival | | 2013 | USA |
| TARGET | Illumina | 0 | 101 | Yes 22  No 65 | MAP-based chemotherapy | Overall survival | | 2019 | USA |
| Total |  | **17** | **306** |  |  |  |  | |  |

**Notes: All specimens were taken from tumor biopsies pre-therapy**
