# Supplementary material for: Dysregulated m6A-Related Regulators Are Associated With Tumor Metastasis and Poor Prognosis in Osteosarcoma
Source: Front Oncol. 2020 Jun 2;10:769. doi: 10.3389/fonc.2020.00769 (PMC7280491; doi:10.3389/fonc.2020.00769)
Supplement: Supplementary file 2 [file Table_2.docx]

**Table S2.** Information on antibodies used in this study

| Antibody | IHC | IF | Specificity | Company |
| --- | --- | --- | --- | --- |
| WTAP | 1:500 | 1:200 | Mouse Monoclonal | Proteintech Group, China |
| KIAA1429 | 1:200 | 1:200 | Rabbit Polyclonal | Proteintech Group, China |
| RBM15 | 1:500 | 1:200 | Rabbit Polyclonal | Proteintech Group, China |
| RBM15B | 1:200 | 1:200 | Rabbit Polyclonal | Proteintech Group, China |
| METTL3 | 1:200 | 1:200 | Rabbit Polyclonal | Proteintech Group, China |
| METTL14 | 1:200 | 1:200 | Rabbit Polyclonal | Proteintech Group, China |
| METTL16 | 1:200 | 1:200 | Rabbit Polyclonal | Proteintech Group, China |
| HNRNPA2B1 | 1:500 | 1:200 | Rabbit Polyclonal | Proteintech Group, China |
| HNRNPC | 1:200 | 1:200 | Rabbit Polyclonal | Proteintech Group, China |
| YTHDF1 | 1:200 | 1:200 | Rabbit Polyclonal | Proteintech Group, China |
| YTHDF2 | 1:200 | 1:200 | Rabbit Polyclonal | Proteintech Group, China |
| YTHDF3 | 1:200 | 1:200 | Rabbit Polyclonal | Proteintech Group, China |
| YTHDC1 | 1:200 | 1:200 | Rabbit Polyclonal | Proteintech Group, China |
| FTO | 1:200 | 1:200 | Rabbit Polyclonal | Proteintech Group, China |
| ALKBH5 | 1:500 | 1:200 | Rabbit Polyclonal | Proteintech Group, China |
