# Supplementary material for: Dysregulated m6A-Related Regulators Are Associated With Tumor Metastasis and Poor Prognosis in Osteosarcoma
Source: Front Oncol. 2020 Jun 2;10:769. doi: 10.3389/fonc.2020.00769 (PMC7280491; doi:10.3389/fonc.2020.00769)
Supplement: Supplementary file 3 [file Table_3.docx]

**Table S3.** **The subcellular location of m6A-related proteins in OS cells.**

| M6A related proteins | Subcellular location | |
| --- | --- | --- |
|  | **Nuclear staining** | **Cytoplasmic staining** |
| WTAP | Intense | Weak |
| KIAA1429 | Intense | Weak |
| RBM15 | Intense | Weak |
| RBM15B | Intense | Weak |
| METTL3 | Intense | Weak |
| METTL14 | Intense | Weak |
| METTL16 | Intense | Weak |
| HNRNPA2B1 | Intense | Unstained |
| HNRNPC | Intense | Unstained |
| YTHDF1 | Weak | Intense |
| YTHDF2 | Weak | Intense |
| YTHDF3 | Intense | Intense |
| YTHDC1 | Weak | Weak |
| FTO | medium | medium |
| ALKBH5 | medium | medium |
