# Supplementary material for: Dysregulated m6A-Related Regulators Are Associated With Tumor Metastasis and Poor Prognosis in Osteosarcoma
Source: Front Oncol. 2020 Jun 2;10:769. doi: 10.3389/fonc.2020.00769 (PMC7280491; doi:10.3389/fonc.2020.00769)
Supplement: Supplementary file 4 [file Image_1.pdf]

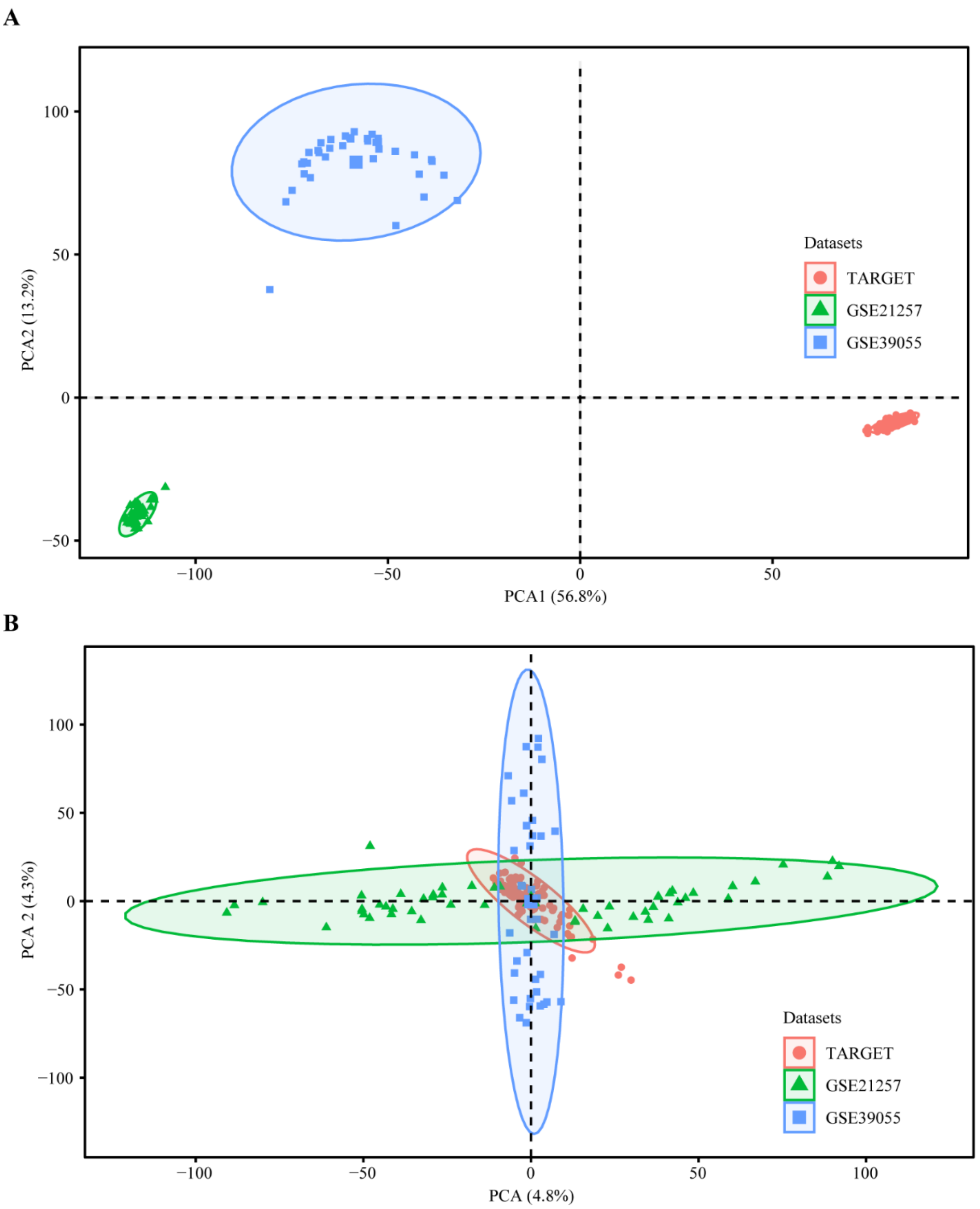

Figure S1. Principal Components Analysis (PCA) of three independent datasets before and after removing batch effect. **(A):** PCA before removing batch effects. **(B):** PCA after removing batch effects.
